# Understanding the Relationship Between Complicated Grief Symptoms and Patterns of Personality Disorders in a Substance Users’ Sample: A Network Analysis Approach

**DOI:** 10.3389/fpsyg.2020.566785

**Published:** 2020-10-30

**Authors:** Laura Masferrer, Anthony D. Mancini, Beatriz Caparrós

**Affiliations:** ^1^CAS Girona, Mental Health and Addiction Research Group, Institutd’Assistència Sanitària (IAS), Institut d’Investigació Biomèdica de Girona (IDIBGI), Girona, Spain; ^2^Department of Psychology, University of Girona, Girona, Spain; ^3^Department of Psychology, Pace University, New York, NY, United States

**Keywords:** personality disorders, substance use disorder, complicated grief, psychopathology, network analysis

## Abstract

**Background:**

The presence of personality disorders is greatly prevalent among substance users. Personality disorders could increase vulnerability to complicated grief symptoms. Bereavement is commonly overlooked among substance users. We used network analysis to estimate the structure of relations between patterns of personality disorders and complicated grief symptoms among a bereaved substance-using population.

**Methods:**

Complicated grief and personality disorders were assessed in a sample of 196 bereaved substance users. We use the graphical least absolute shrinkage selection operator (LASSO) to compute a regularized partial correlation network revealing associations among different patterns of personality disorders and complicated grief symptoms.

**Results:**

In a network involving nodes for personality disorders and symptomatology of complicated grief, patterns of depressive and paranoid personality disorder showed small relationships to complicated grief symptoms. All other personality disorders showed negligible to no relationship to complicated grief symptoms. Further, in the overall network, complicated grief showed the lowest level of centrality, suggesting that it is independent of personality disorders, whereas depressive and paranoid personality disorder symptoms showed the highest centrality.

**Conclusion:**

Network analysis can be used to understand the relationships among higher-level constructs such as disorders. We found that complicated grief is largely independent of patterns of personality disorders with the exception of depressive and paranoid. Findings have implications for assessment and appropriate treatment of complicated grief symptoms and substance use disorder.

## Introduction

The majority of people adapt to bereavement over time (Bonanno et al., 2011), but for a minority, grief symptoms remain elevated and persistent, which is a clinical syndrome often described as complicated grief (Zhang et al., 2006; Boelen and van den Bout, 2008). Different studies have found a prevalence of complicated grief symptoms in about 10–20% (Lobb et al., 2010; Mancini and Bonanno, 2012; Lundorff et al., 2017). The 11th edition of the International Classification of Diseases (ICD) included complications in bereavement as “prolonged grief disorder,” characterized by core symptoms such as longing for and preoccupation with the deceased, along with emotional distress and significant functional impairment that persist beyond half a year after the loss of a significant other (World Health Organization, 2018). People with substance use disorders (SUDs) are particularly likely to have faced significant adversity. People with SUD frequently report life stories marked by suffering, traumatic experiences during childhood (Cuomo et al., 2008), economic instability, unemployment (Sumnall and Brotherhood, 2012), physical complications (Sanchez-Peña et al., 2012), as well as social exclusion (Calabria et al., 2010). Moreover, people with SUD are a particularly vulnerable subpopulation and report high levels of trauma and loss experiences (Martin and Privette, 1989; Farley et al., 2004; Zuckoff et al., 2006; Furr et al., 2015). However, grief symptoms are frequently ignored among patients with SUDs, despite their traumatic background. Indeed, the antecedents of complicated grief among people with SUD people are not well understood. One factor that may increase vulnerability to complicated grief is personality disorders (PD), which have a well-documented association with SUD (Trull et al., 2010; Goldstein et al., 2012; Agrawal et al., 2013). People with PD could have more intense reactions to the loss of a significant person than people without a personality disorder diagnosis (Giourou et al., 2018). Moreover, personality disorders enhance the likelihood of feelings of abandonment and rejection sensitivity that could impair adaptation to bereavement (Brüne, 2016; Millon, 2016). Although some prior research has examined the relation between personality disorders and complicated grief, no prior research has used network analysis to explore the relationships among these constructs.

Personality disorder is conceptualized as “an enduring pattern of inner experience and behavior that deviates markedly from the expectations of the individual’s culture, is pervasive and inflexible, has an onset in adolescence or early adulthood, is stable over time, and leads to distress or impairment” (American Psychiatric Association, 2013). According to the *Diagnostic and Statistical Manual of Mental Disorders* (*DSM*) (American Psychiatric Association, 2013), personality disorders from different clusters frequently co-occur. Although personality disorders have historically been treated as categorical entities (American Psychiatric Association, 2013), there is increasing recognition that personality disorders are more appropriately viewed as dimensional and overlapping. In this approach, personality disorders are defined by impairments in personality functioning, which vary in severity across different personality disorder diagnoses (Widiger and Trull, 2007; Eaton et al., 2010). In support of this perspective, subthreshold disorder manifestations are linked with significant distress and dysfunction, indicating an underlying dimensionality to mental disorders not captured by categorical diagnoses (Krueger and Eaton, 2015).

The presence of personality disorders is widely prevalent among SUDs (Jahng et al., 2011; Langås et al., 2012; Walcott et al., 2013; Casadio et al., 2014). Prevalence estimates for the different personality disorders in a sample of substance abusers suggest 42.6%; cluster B was the most common, followed by clusters C and A (Colpaert et al., 2012). In fact, this study is part of wider research in which the presence of any personality disorders assessed with Millon Clinical Multiaxial Inventory (MCMI-III, Millon et al., 2007) was 29.4% (Masferrer and Caparrós, 2017). Moreover, there is some degree of overlap between personality disorders and SUD criteria, which raises the possibility that some comorbidity could be a consequence of definitional overlap (Sher and Trull, 2002). Given the dimensional nature of personality disorders and their overlap with SUD, the analysis of simple, unidirectional relationships between personality disorders and other constructs ignores the potential for conceptual overlap and reciprocal relationships (McNally et al., 2017).

There are a number of reasons why personality disorders could increase risk for complicated grief symptoms. Personality disorders index a wide range of maladaptive emotional, cognitive, and behavioral traits. For example, personality disorders are associated with affective instability, a fluctuating sense of identity, and maladaptive coping behaviors (Hopwood et al., 2012). Interpersonal dependency, which is implicated in various personality disorders, has shown a link with maladaptive grief outcomes (Denckla et al., 2011). In the context of loss, these maladaptive traits could have particularly deleterious effects, increasing risk for complicated grief symptoms.

Moreover, it has been widely shown that personality disorders are associated with greater risk of dysfunctional reactions to traumatic events. Specifically, patients with comorbidity between SUD and post-traumatic stress disorder (PTSD) have been found to have a more severe clinical profile in comparison to patients with either SUD or PTSD alone (Hoorelbeke et al., 2016). Comorbidity of SUD and PTSD is specifically associated with a greater incidence of personality disorders (Schäfer and Najavits, 2007). Consistent with this relation, some studies have found that difficulties in emotional regulation are associated with PTSD (Ehring and Quack, 2010; Roberts et al., 2015), and it is important to note that many personality disorders have emotional dysregulation as a nuclear symptom. This is particularly relevant in the case of histrionic and narcissistic personality disorder. Narcissistic vulnerability was found as a contributor to the occurrence of PTSD (Bachar et al., 2005). Using a psychodynamic approach, Horowitz (1999) argued that people with hysterical and obsessive patterns were more vulnerable to complications in bereavement mainly because of a biased interpretation of the meaning of loss. However, empirical findings have yet to bear this supposition out. For example, Tomarken et al. (2001) found that obsessive, histrionic, and narcissistic personality patterns were not associated with complicated grief symptoms. In the present study, therefore, we used a network analysis to examine complex associations between patterns of personality disorders and complicated grief symptoms.

The current research is based on Millon’s integrative model of personality disorders (Millon, 2011), which is derived from evolutionary theory and uses different perspectives (biological, interpersonal, cognitive, and psychodynamic). Millon classifies personality disorders along four main dimensions: personalities with difficulties in taking pleasure (i.e., avoidant, schizoid, or depressive disorders), personalities with interpersonal problems (i.e., dependent, histrionic, narcissistic, or antisocial disorders), personalities with intrapsychic conflict (i.e., compulsive, sadistic, masochistic, or negativistic disorders), and personalities with structural deficits (i.e., schizotypal, borderline, and paranoid disorders) (Millon, 2011). One key aspect of Millon’s conceptualization is the proposal that personality disorders are embedded in how people understand themselves and relate to others, a “self–other” polarity that aligns with interpersonal problems (Millon, 2011). Given that dependency is associated with complicated grief (Denckla et al., 2011; Mancini et al., 2015), this suggests that personality disorders that involve interpersonal problems could increase risk for complicated grief symptoms. In addition, because personality disorders involve structural deficits that could lead to an inability to represent the self, interpersonal loss could be particularly detrimental for people who rely on others for their self-understanding. Indeed, conceptualizations of complicated grief increasingly suggest links between the dynamics of identity, feelings of dependency, and the risk for complicated grief (Maccallum and Bryant, 2013). These factors suggest potential links between complicated grief symptoms and personality disorder that may have eluded prior research, because of their focus on *DSM* conceptualizations of personality disorder.

Network analysis defines mental disorders as a causal system of functionally interrelated symptoms that have assumed a pathological equilibrium (Blumenfeld et al., 2004). In the network approach, each symptom is represented by a node, while the edge between two nodes represents the relationship between them. Prior research on networks of grief and depression symptoms suggested a clear relationship between the two disorders, with loneliness serving as a bridge symptom (Borsboom and Cramer, 2013; Robinaugh et al., 2014; McNally et al., 2015; Fried et al., 2017). Indeed, previous network analyses have focused exclusively on networks of symptoms, arguing that symptoms are “mereological” and constitute the disorder itself. This approach calls into question the idea that the symptoms of grief are the result of an unobservable or latent construct, such as complicated grief (Butte and Kohane, 1999). However, the network approach can also be used to decipher the interrelationships among higher-order constructs (Millon et al., 1997; Hoorelbeke et al., 2016) in order to obtain a fine-grained understanding of their centrality and possible directionality (Borsboom and Cramer, 2013; Costantini et al., 2015; Hoorelbeke et al., 2016), including examining relationships among diagnostic entities. Because no prior studies have estimated the relation between personality disorders and complications in bereavement, we addressed this question broadly and at a construct level. After determining the network of relations among complicated grief symptoms and personality disorders, we explored pairwise interactions among symptoms specifically.

In the present study, we sought to better understand the relationships among patterns of personality disorders and complicated grief symptoms among an SUD population. We used a network analysis to uncover these relationships and to provide a visual depiction of these associations, which can help to illuminate theoretical links among complicated grief symptoms and personality disorders and may inform treatment approaches for symptomatology of complicated grief.

## Materials and Methods

### Participants

This study is part of a wider research program on complicated grief and substance use disorders (Masferrer and Caparrós, 2017). The current research was based on a consecutive non-probabilistic sampling of convenience.

Participants were outpatients of an addiction treatment center. The inclusion criteria were (1) diagnosis of alcohol, cocaine, or heroin dependence according to the *DSM-IV-TR*, (2) suffering the loss of a significant person (family, best friend, or partner) at least a year previously and at any time during their life, and (3) abstinence during the last month. The sample size was calculated on the basis of the estimated prevalence of complicated grief symptoms in the general population and an assumed prevalence of 15% with an alpha level of 0.05 for a precision of ±0.05. For the present study, we interviewed 205 patients, but 9 participants were excluded for the current analysis because they did not meet the inclusion criteria, resulting in a final sample of 196. Participants were, on average, 45.58 years old (SD = 10.14) and largely male (78.1%), working (36.7%), with secondary studies (67%). Most were married or with a partner (37.3%), followed by divorced or separated (32.1%), with the remaining single (22.4%) or widowed (8.2%). Previous articles reported the following information.

### Measures

Personality disorders were assessed using the Spanish version of the *Millon Clinical Multiaxial Inventory* (Millon et al., 1997; Cardenal and Sánchez-López, 2007). The MCMI-III is a 175-item, dichotomous-answer (true/false), self-report questionnaire that measures 11 clinical personality patterns (schizoid, avoidant, depressive, dependent, histrionic, narcissistic, antisocial, aggressive, compulsive, negativistic, and self-destructive), 3 traits of severe personality pathology that represents advanced states of personality pathology (schizotypal, borderline, and paranoid), 7 clinical syndromes of moderate severity (anxiety disorder, somatic disorder, bipolar disorder, dysthymic disorder, alcohol dependence, substance dependence, PTSD), 3 severe clinical syndromes (thought disorder, major depression, delusional disorder), and a validity scale and 3 modifying indices (disclosure scale, desirability scale, and debasement scale). The PD scales cover major diagnostic criteria of the *DSM-IV*. Cronbach’s alpha ranged from 0.66 to 0.80, and the test–retest reliability ranged from 0.85 to 0.93. The test–retest reliability for the categorical diagnosis was moderate, *k* < 0.45.

Complicated grief symptoms were assessed using the Spanish version of the *Inventory of Complicated Grief* (ICG) (Limonero et al., 2009). It consists of 19 items. Responses are provided on a five-point Likert scale representing an increase in severity (0—never, 1—seldom, 2—sometimes, 3—often, and 4—always) (range = 0–76). To determine a complicated grief diagnosis, we used a cutoff point of 25 based on the English version of the ICG (Prigerson et al., 1995). The internal consistency of the Spanish version was high at 0.88 Cronbach’s alpha and presented at 0.81 test–retest reliability.

### Procedure

Participants who met the three inclusion criteria were notified by their therapist reference. If patients agreed to collaborate, the psychologist (who is the first author) called each patient to plan an appropriate time for an interview for them. All participants were informed about study procedures as well as terms of confidentiality. The psychometric tests were conducted following the Organic Law 15/1999 of Protection of Personal Data. Informed consent was obtained from all participants, and the protocol was approved by the Institutional Ethics and Research Review Board of the Institut Assistència Sanitària (IAS).

### Statistical Analysis

For personality disorders as well as for complicated grief symptoms, we used continuous variables as the basis for our analyses. To visualize networks, we used the *R* package *qgraph* (Epskamp et al., 2012). R package bootnet allowed us to estimate psychological networks in a generalized framework. We implemented glasso in combination with Extended Bayesian Information Criteria (EBIC) model selection to estimate regularized Gaussian Graphical Model (GGM) nodes (Friedman et al., 2014; Rhemtulla et al., 2016; Epskamp et al., 2018).

To determine the role of personality disorders and symptoms of complicated grief in the network, we calculated centrality indices. Centrality can be understood to reflect how linked the different constructs are and how potentially clinically relevant a construct is in a network (Fonseca-Pedrero, 2018). Network models make the assumption that the pattern of relations among constructs is direct, bidirectional causal pathways among variables, so intervening on a highly central construct will affect other nodes both directly and indirectly, pushing the entire network into a healthier state (Borsboom and Cramer, 2013). To determine the centrality of personality disorder and complicated grief symptoms in our networks, we first focused on the strength metric (Opsahl et al., 2010) because of its theoretical relevance to and reliability in psychopathology networks (Fried et al., 2018). Strength reflects the sum of the absolute value of edge weights for a given node (regularized partial correlations). An extension of the strength metric is expected influence (EI) (Robinaugh et al., 2016). EI calculates the sum of edge weights, but it also retains the negative value (or sign) of the weight. EI is identical to the strength index when there are no negative edges but can be substantially different when there are negative edges. EI provides a more accurate index of node centrality when negative edges are present (Robinaugh et al., 2016).

## Results

### Psychopathological Variables

More than a half of participants had a diagnosis of alcohol dependence (68.9%), 18.4% had heroin dependence, and 12.8% had cocaine dependence. Related to complications in bereavement, 34.2% of patients met criteria for complicated grief symptoms (Masferrer et al., 2017). The occurrence of any personality disorders in the sample was 29.4%. The personality disorders with higher frequency were compulsive (7.1%) and narcissistic (7.1%) followed by antisocial (4.6%) and sadistic (3.1%) (Masferrer and Caparrós, 2017).

### Network Analysis

**FIGURE 1:**
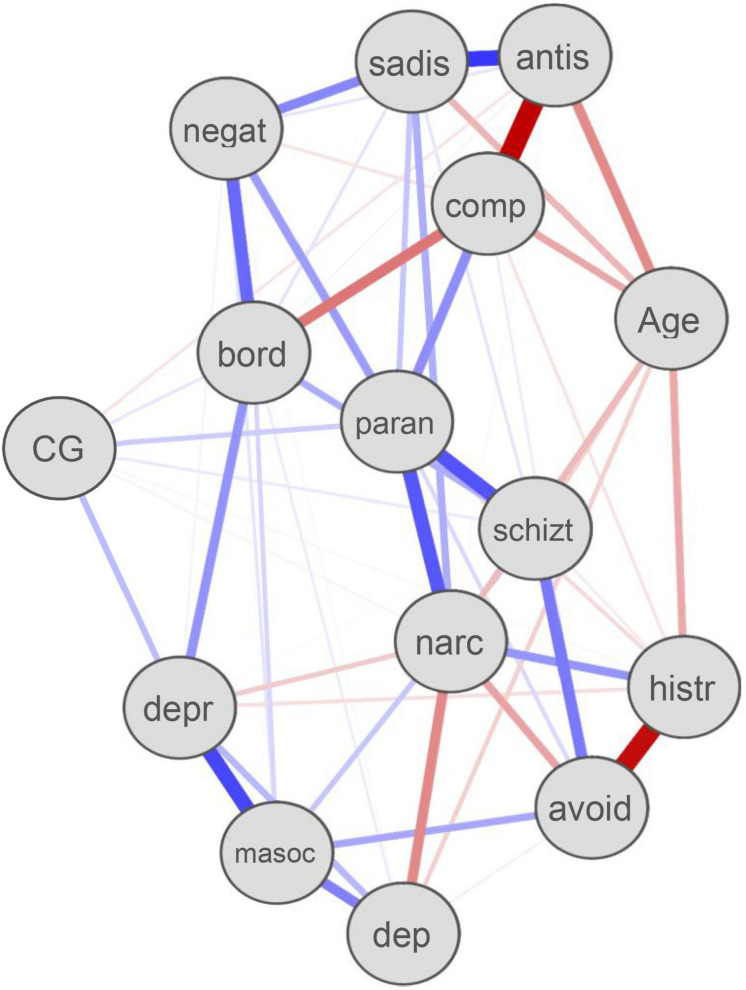
Personality disorders and complicated grief symptoms network. Paran, paranoid; negat, negativistic; narc, narcissistic; bord, borderline; CG, complicated grief; masoc, self-destructive; sadis, sadistic or aggressive; comp, compulsive; antis, antisocial; dep, dependent; avoid, avoidant; histr, histrionic; schizt, schizotypal; depr, depressive.

The results of the estimated network analysis are presented in Figure 1. Thicker edge weights represent stronger partial correlations, controlling for all other correlations. Blue edges represent positive relations, and red edges represent negative relations. These are partial correlation coefficients. Nodes that are closer together are more strongly related. Although complicated grief symptoms were associated with patterns of depressive (edge = 0.15), paranoid (edge = 0.12), schizotypal (edge = 0.05), and borderline (edge = 0.05), the network edges were weak. In addition, most patterns of personality disorder showed no relation to complicated grief symptoms, including patterns of avoidant, schizoid, negativistic, sadistic, and masochistic. Interestingly, patterns of narcissistic (edge = −0.02), histrionic (edge = −0.02), and antisocial (edge = −0.06) showed small negative relations to complicated grief symptoms.

**FIGURE 2:**
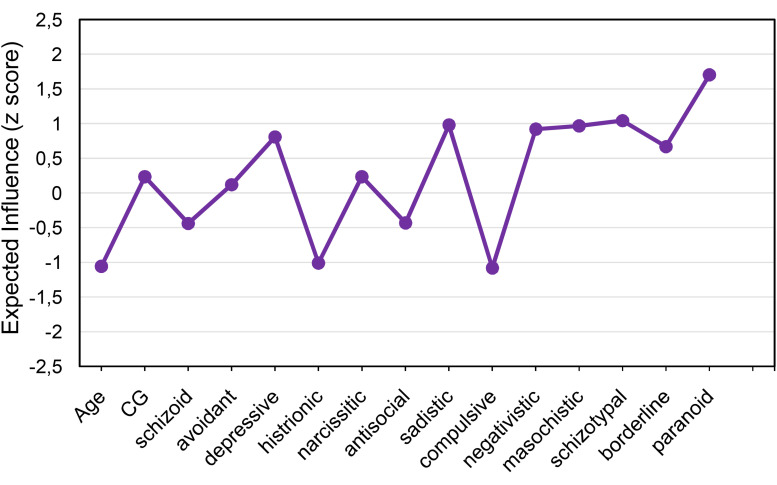
Expected influence for each psychological construct.

*Network Centrality*. Because negative edges were present in our LASSO networks, we relied on EI as our measure of centrality (Robinaugh et al., 2016). Complicated grief is the second least central construct and reveals low EI (0.24) (Figure 2), suggesting that it contributes little to the network. Interestingly, the pattern of avoidant personality disorder also showed the lowest degree of EI (0.12), suggesting that it exerts less influence on the overall network of personality disorder symptoms. The pattern of personality disorder nodes with the strongest influence on the network was paranoid (EI = 1.71).

## Discussion

The main objective of this study was to identify relationships among patterns of personality disorders and complicated grief symptoms among a bereaved SUD population. To our knowledge, the present study is the first to use network analysis to estimate the relation between patterns of personality disorders and complicated grief symptoms. Using a network approach focused at the disorder level, we found that complicated grief symptoms showed relatively few relationships to patterns of personality disorders. However, a number of personality disorders were strongly related to one another, though in some cases, these relationships were negative. This finding is consistent with the high degree of comorbidity of personality disorders but also suggests underlying dimensions that are inversely related. Together the results suggest that complicated grief symptoms are not a consequence of underlying personality disorder.

The present findings contribute to a modest literature on bereavement and personality disorder. Although several studies have examined personality disorders among SUD samples (Goldstein et al., 2012; Langås et al., 2012; Agrawal et al., 2013), relatively little research has examined personality disorders and complicated grief symptoms among an SUD population. Overall, complicated grief symptoms showed relatively few and weak edge weights with specific patterns of personality disorder symptoms. These edge weights represent partial correlation coefficients and can be interpreted as effect size in terms of traditional conventions for magnitude (Cohen, 1992). On this basis, the relations between complicated grief symptoms and patterns of schizotypal, compulsive, borderline, and paranoid were negligible to very small in terms of their magnitude (edges from 0.01 to 0.11). Both narcissistic and antisocial personality disorder showed negative edges with complicated grief symptoms, indicating that they show small negative associations with grief (edges −0.03 and −0.08, respectively). The only pattern of MCMI-III personality disorder to show a modest relationship to complicated grief symptoms was depressive (edge = 0.15), a result consistent with prior research. As Robinson and Marwit (2006) stated, bereaved mothers with higher levels of distress following loss showed high levels of neuroticism characterized by guilt, low self-esteem, irrationality, shyness, and moodiness. Moreover, some neurotic personality traits that reflect difficulties in emotional regulation are prone to complicated grief symptoms (Prigerson et al., 1997). The current results are consistent with previous finding that histrionic and narcissistic personality disorders show little relation to complicated grief symptoms (Tomarken et al., 2001). Some personality traits from personality disorders are adaptive as long as they do not take an extreme form (Prigerson et al., 1997). In support of this line of reasoning, previous research carried out with the MCMI found that a curvilinear function characterized the histrionic and compulsive scales (Millon et al., 2007). This implied that it is the low and the high scores that are maladaptive, whereas intermediate levels on these scales would indicate adaptive patterns (Caparrós and Villar, 2013).

In the present study, we found little evidence that patterns of personality disorder are potential risk factors in bereavement (Meuser and Marwit, 2000) and that on balance, complicated grief is not a consequence of personality disorder psychopathology.

Consistent with the findings on edge weights, we also found that complicated grief symptoms had low levels of centrality in the overall network. This further suggests that complications in bereavement are not embedded in personality disorder psychopathology. Rather, complicated grief likely reflects other factors related to the experience of loss itself. The one exception is the relation of complicated grief symptoms to the pattern of depressive personality disorder. This suggest that treatments should focus on the individual’s distinctive experience of loss and not on dispositional characteristics, especially among those with comorbid SUDs, who appear to be at greater risk for grief symptoms.

These findings also support the high levels of comorbidity typically found in personality disorders (Pfohl et al., 1986). However, it should also be noted that there were a considerable number of negative edges between patterns of personality disorder, suggesting the presence of underlying dimensions that are negatively interacting, with one tending to suppress another dimension. In fact, comorbidity might be understood as an artifact of the diagnostic system (Bonanno, 1999). In relation to centrality, which summarizes the likelihood that a construct is central to the overall network, the patterns of depressive as well as paranoid personality disorder were the most central. The dimensional structure of the pattern of paranoid personality disorder is highly relevant (Edens et al., 2009) because this may represent one that transcends other personality disorders (Bernstein and Useda, 2007). There results offer some support for Millon’s suggestion that patterns of paranoid personality disorders are more maladaptive components of personality pathology (Millon, 2011), insofar as each of these had the highest levels of centrality to the overall network.

The present findings further suggest that substance use treatment should bear in mind the relevance of complicated grief symptoms. It is particularly noteworthy that a depressive personality pattern, characterized by being prone to negative affect, self-criticism, low self-esteem, and pessimistic outlook, may be a marker of complicated grief symptoms, and as premorbid personality can affect the grieving process, treatments for substance use that consider the simultaneous role of this pattern and grief would be a particular benefit.

This study has a number of limitations. First, the use of a self-report scale may yield socially desirable responding. The present research is cross-sectional; therefore, it does not permit the testing of temporal relationships among the variables, and causal inferences cannot be made. Because of the small sample size, our findings may not be replicated and should be understood as generating hypotheses for future research. Notwithstanding the relatively small sample, this work offers valuable insights into the associations between patterns of personality disorders and symptoms of complicated grief. Future research should focus on the symptom level as opposed to the disorder level, in order to better understand the connections between specific complicated grief symptoms and personality disorder symptoms and their bidirectional influence. Specific complicated grief symptoms (for example, loneliness) may bear a particular relation to substance use, and this would be a useful topic for future research.

Up to now, far too little attention has been paid to personality disorders and bereavement. To our knowledge, this is the first study to estimate patterns of personality disorders and complicated grief symptoms among an SUD sample through network analysis. Given that complicated grief played a weak role in a network of patterns of personality disorders, our findings underscore the independent nature of grief-related pathology (Bonanno et al., 2007). Although grief is a normative experience (Mancini and Bonanno, 2009; Zimmermann et al., 2014), complicated grief has significant negative effects on functioning. Complicated grief symptoms among substance users are likely underappreciated, and interventions for substance use should bear in mind that depressive personality symptoms may enhance the risk of complicated grief symptoms.

## Data Availability Statement

The raw data supporting the conclusions of this article will be made available by the authors, without undue reservation.

## Ethics Statement

The studies involving human participants were reviewed and approved by the Institutional Ethics and Research Review Board of Institut Assistència Sanitària (IAS). The patients/participants provided their written informed consent to participate in this study.

## Author Contributions

LM and BC conceived and designed the research. LM collected field data and wrote the manuscript. AM guided the statistical analysis. AM revised the manuscript. All authors interpreted the results and approved the final manuscript. All authors contributed to the article and approved the submitted version.

## Conflict of Interest

The authors declare that the research was conducted in the absence of any commercial or financial relationships that could be construed as a potential conflict of interest.

## References

[B1] Agrawal A. Narayanan G. Oltmanns T. (2013). Personality pathology and alcohol dependence at midlife in a community sample. *J. Pers. Disord.* 4 55–61. 10.1037/a0030224 23230852 PMC3575957

[B2] American Psychiatric Association (2013). *Diagnostic and Statistical Manual of Mental Disorders*, 5 Edn Washington, DC: APA.

[B3] Bachar E. Hadar H. Shalev A. (2005). Narcissistic Vulnerability and the Development of PTSD. *J. Nerv. Ment. Dis.* 193 762–765. 10.1097/01.nmd.0000185874.31672.a5 16260935

[B4] Bernstein D. P. Useda J. (2007). “Paranoid personality disorder,” in *Personality disorders: Toward the DSM-V*, eds O’Donohue W. Fowler K. Lilienfeld S. (Thousand Oaks, CA: Sage), 41–62. 10.1037/11476-003

[B5] Blumenfeld H. McNally K. A. Vanderhill S. D. Paige A. L. Chung R. Davis K. (2004). Positive and negative network correlations in temporal lobe epilepsy. *Cereb. Cortex* 14 892–902. 10.1093/cercor/bhh048 15084494

[B6] Boelen P. van den Bout J. (2008). Complicated grief and uncomplicated grief are distinguishable constructs. *Psychiatry Res.* 157 311–314. 10.1016/j.psychres.2007.05.013 17916387

[B7] Bonanno G. (1999). “Factors associated with effective loss accommodation. Conceptual, theoretical, and treatment foundations,” in *Traumatology of Grieving. Philadelphia*, ed. Figley C. R. (New York, NY: Brunner- Mazel), 37–52.

[B8] Bonanno G. Westphal M. Mancini A. (2011). Resilience to loss and potential trauma. *Annu. Rev. Clin. Psychol.* 7 511–535. 10.1146/annurev-clinpsy-032210-104526 21091190

[B9] Bonanno G. A. Neria Y. Mancini A. Coifman K. G. Litz B. Insel B. (2007). Is there more to complicated grief than depression and posttraumatic stress disorder? A test of incremental validity. *J. Abnorm. Psychol.* 116:342. 10.1037/0021-843x.116.2.342 17516766

[B10] Borsboom D. Cramer A. (2013). Network analysis: an integrative approach to the structure of psychopathology. *Annu. Rev. Clin. Psychol.* 9 91–121. 10.1146/annurev-clinpsy-050212-185608 23537483

[B11] Brüne M. (2016). Borderline personality disorder: why ‘fast and furious’? *Evol. Med. Public Health* 2016 52–66. 10.1093/emph/eow002 26929090 PMC4782519

[B12] Butte A. J. Kohane I. S. (1999). Unsupervised knowledge discovery in medical databases using relevance networks. *Proc. AMIA Symp.* 1999 711–715. PMC2232846 10566452

[B13] Calabria B. Degenhardt L. Briegleb C. Vos T. Hall W. McLaren J. (2010). Systematic review of prospective studies investigating “remission” from amphetamine, cannabis, cocaine or opioid dependence. *Addict. Behav.* 35 741–749. 10.1016/j.addbeh.2010.03.019 20444552

[B14] Caparrós B. Villar E. (2013). Millon clinical multiaxial inventory III (MCMI-III) and communication styles in a sample of university students. *Span. J. Psychol.* 16 1–12. 10.1017/sjp.2013.85 24230948

[B15] Cardenal V. Sánchez-López M. P. (2007). *Manual MCMI-III. Adaptación y Baremación Españolas.* Madrid: TEA Ediciones.

[B16] Casadio P. Olivoni D. Ferrari B. Pintori C. Speranza E. Bosi M. (2014). Personality disorders in addiction outpatients: prevalence and effects on psychosocial functioning. *J. Subst. Abuse* 8 17–24. 10.4137/SART.S13764 24701119 PMC3972129

[B17] Cohen J. (1992). A power primer. *Psychol. Bull.* 112 155–159. 19565683 10.1037//0033-2909.112.1.155

[B18] Colpaert K. Vanderplasschen W. De Maeyer J. Broekaert E. De Fruyt F. (2012). Prevalence and determinants of personality disorders in a clinical sample of alcohol-, drug-, and dual- dependent patients. *Subst. Use Misuse* 47 649–661. 10.3109/10826084.2011.653427 22288949

[B19] Costantini G. Richetin J. Borsboom D. Fried E. Rhemtulla M. Perugini M. (2015). Development of indirect measures of conscientiousness: combining a facets approach and network analysis. *Eur. J. Pers.* 29 548–567. 10.1002/per.2014

[B20] Cuomo C. Sarchiapone M. Di Giannantonio M. Mancini M. Roy A. (2008). Aggression, impulsivity, personality traits, and childhood trauma of prisoners with substance abuse and addiction. *Am. J. Drug Alcohol Abuse* 34 339–345. 10.1080/00952990802010884 18428076

[B21] Denckla C. Mancini A. Bornstein R. Bonanno G. (2011). Adaptive and maladaptive dependency in bereavement: distinguishing prolonged and resolved grief trajectories. *Pers. Indiv. Differ.* 51 1012–1017. 10.1016/j.paid.2011.08.014 21984858 PMC3188454

[B22] Eaton N. R. South S. C. Krueger R. F. (2010). “The meaning of comorbidity among common mental disorders,” in *Contemporary Directions in Psychopathology*, 2nd Edn, eds Millon T. Krueger R. Simonsen E. (New York: Guilford Press), 223–241.

[B23] Edens J. F. Marcus D. K. Morey L. C. (2009). Paranoid personality has a dimensional latent structure: taxometric analyses of community and clinical samples. *J. Abnorm. Psychol.* 118 545–553. 10.1037/a0016313 19685951 PMC2853919

[B24] Ehring T. Quack D. (2010). Emotion regulation difficulties in trauma survivors: the role of trauma Type and PTSD symptom severity. *Behav. Ther.* 41 587–598. 10.1016/j.beth.2010.04.004 21035621

[B25] Epskamp S. Borsboom D. Fried E. (2018). Estimating psychological networks and their accuracy: a tutorial paper. *Behav. Res.* 50 195–212. 10.3758/s13428-017-0862-1 28342071 PMC5809547

[B26] Epskamp S. Cramer A. O. J. Waldorp L. J. Schmittmann V. D. Borsboom D. (2012). Qgraph: network visualizations of relationships in psychometric data. *J. Stat. Softw.* 48 1–18.

[B27] Farley M. Golding J. Young G. Mulligan M. Minkoff J. (2004). Trauma history and relapse probability among patients seeking substance abuse treatment. *J. Subst. Abuse Treat.* 27 161–167. 10.1016/j.jsat.2004.06.006 15450649

[B28] Fonseca-Pedrero E. (2018). Network analysis in psychology. *Psychol. Pap.* 39 1–12.

[B29] Fried E. Eidhof M. B. Palic S. Costantini G. Huisman-van Dijk H. M. Bockting C. L. H. (2018). Replicability and generalizability of PTSD networks: a cross-cultural multisite study of PTSD symptoms in four trauma patient samples. *Clin. Psychol. Sci.* 6 335–351. 10.1177/2167702617745092 29881651 PMC5974702

[B30] Fried E. van Borkulo C. Cramer A. Boschloo L. Schoevers R. Borsboom D. (2017). Mental disorders as networks of problems: a review of recent insights. *Soc. Psychiatry Psychiatr. Epidemiol.* 52 1–10. 10.1007/s00127-016-1319-z 27921134 PMC5226976

[B31] Friedman J. H. Hastie T. Tibshirani R. (2014). *Glasso: Graphical Lasso- Estimation of Gaussian Graphical Models. R Package Version 1.8.*

[B32] Furr S. Johnson W. Goodall C. (2015). Grief and recovery: the prevalence of grief and loss in substance abuse treatment. *J. Addict. Offend. Counsel.* 36 43–56. 10.1002/j.2161-1874.2015.00034.x

[B33] Giourou E. Skokou M. Andrew S. P. Alexopoulou K. Gourzis P. Jelastopulu E. (2018). Complex posttraumatic stress disorder: the need to consolidate a distinct clinical syndrome or to reevaluate features of psychiatric disorders following interpersonal trauma? *World J. Psychiatry* 8 12–19. 10.5498/wjp.v8.i1.12 29568727 PMC5862650

[B34] Goldstein R. Dawson D. Chou S. Grant B. (2012). Sex differences in prevalence and comorbidity of alcohol and drug use disorders: results from wave 2 of the national epidemiologic survey on alcohol and related conditions. *J. Stud. Alcohol Drugs* 73 938–950. 10.15288/jsad.2012.73.938 23036212 PMC3469048

[B35] Hoorelbeke K. Marchetti I. De Schryver M. Koster E. (2016). The interplay between cognitive risk and resilience factors in remitted depression: a network analysis. *J. Affect. Disord.* 195 96–104. 10.1016/j.jad.2016.02.001 26878206

[B36] Hopwood C. J. Thomas K. M. Markon K. E. Wright A. G. C. Krueger R. F. (2012). DSM-5 personality traits and DSM–IV personality disorders: correction to Hopwood et al. (2012). *J. Abnorm. Psychol.* 121:432 10.1037/a0028553 PMC3909514 22250660

[B37] Horowitz M. J. (1999). *Essential Papers in Posttraumatic Stress Disorder.* New York, NY: New York University Press.

[B38] Jahng S. Trull T. Wood P. Tragesser S. Tomko R. Grant J. (2011). Distinguishing general and specific personality disorder features and implications for substance dependence comorbidity. *J. Abnorm. Psychol.* 120 656–669. 10.1037/a0023539 21604829 PMC4241053

[B39] Krueger R. Eaton N. (2015). Transdiagnostic factors of mental disorders. *World Psychiatry* 14 27–29. 10.1002/wps.20175 25655146 PMC4329885

[B40] Langås A. Malt U. Opjordsmoen S. (2012). In-depth study of personality disorders in first- admission patients with substance use disorders. *BMC Psychiatry* 12:180. 10.1186/1471-244X-12-180 23107025 PMC3514215

[B41] Limonero J. Lacasta M. García J. Maté J. Prigerson H. (2009). Adaptación al castellano del inventario de duelo complicado. *Med. Paliativa* 16 291–297. 10.4321/s1887-85712011000400006 31832788

[B42] Lobb E. Janson K. Aoun S. Monterosso L. Halkett G. Davies A. (2010). Predictors of complicated grief: a systematic review of empirical studies. *Death Stud.* 34 673–698. 10.1080/07481187.2010.496686 24482845

[B43] Lundorff M. Holmegren H. Zachariae R. Farver-Vestergaard I. O’Connor M. (2017). Prevalence of prolonged grief disorder in adult bereavement: a systematic review and meta-analysis. *J. Affect. Disord.* 212 138–140. 10.1016/j.jad.2017.01.030 28167398

[B44] Maccallum F. Bryant R. (2013). A cognitive attachment model of prolonged grief: integrating attachments, memory, and identity. *Clin. Psychol. Rev.* 33 713–727. 10.1016/j.cpr.2013.05.001 23792468

[B45] Mancini A. D. Bonanno G. A. (2009). Predictors and parameters of resilience to loss: towards an individual differences model. *J. Pers.* 77 1805–1832. 10.1111/j.1467-6494.2009.00601.x 19807863 PMC4224188

[B46] Mancini A. D. Bonanno G. A. (2012). The persistence of attachment: complicated grief, threat, and reaction times to the deceased’s name. *J. Affect. Disord.* 125 316–322. 10.1016/j.jad.2012.01.032 PMC3489169 22387054

[B47] Mancini A. D. Sinan B. Bonanno G. A. (2015). Predictors of prolonged grief, resilience, and recovery among bereaved spouses. *J. Clin. Psychol.* 71 1245–1258. 10.1002/jclp.22224 26394308

[B48] Martin S. Privette G. (1989). Process model of grief therapy in an alcohol treatment program. *J. Spec. Group Work* 14 46–52. 10.1080/01933928908411886

[B49] Masferrer L. Caparrós B. (2017). Risk of suicide and Personality disorders among substance users. *Int. J. Environ. Res. Public Health* 14:316. 10.3390/ijerph14030316 28335530 PMC5369152

[B50] Masferrer L. Garre-Olmo J. Caparrós B. (2017). Is complicated grief a risk factor for substance use? A comparison of substance-users and normative grievers. *Addict. Res. Theory* 25 361–367. 10.1080/16066359.2017.1285912

[B51] McNally R. J. Heeren A. Robinaugh D. J. (2017). A Bayesian network analysis of posttraumatic stress disorder symptoms in adults reporting childhood sexual abuse. *Eur. J. Psychotraumatol.* 8 1–10. 10.1017/s0033291720001750 29038690 PMC5632780

[B52] McNally R. J. Robinaugh D. Wu G. Wang L. Deserno M. Borsboom D. (2015). Mental disorders as causal systems. *Clin. Psychol. Sci.* 3 836–849.

[B53] Meuser T. M. Marwit S. J. (2000). An integrative model of personality, coping and appraisal for the prediction of grief involvement in adults. *Omega* 40 375–393. 10.2190/p6bm-qu6c-6xy9-bnum 22612255

[B54] Millon T. (2011). Classifying personality disorders: an evolution-based alternative to an evidence-based approach. *J. Pers. Disord.* 25 279–304. 10.1521/pedi.2011.25.3.279 21699392

[B55] Millon T. (2016). What is personality disorder? *J. Pers. Disord.* 30 289–306. 10.1521/pedi.2016.30.3.289 27243919

[B56] Millon T. Davis R. Millon C. (1997). *Millon Clinical Multiaxial Inventory-III.* Minneapolis, MN: NCS Pearson.

[B57] Millon T. Davis R. Millon C. Cardenal Hernáez V. Sánchez López M. (2007). *Inventario Clínico MultiaxialMillon. MCMI-III.* Madrid: TEA.

[B58] Opsahl T. Agneessens F. Skvoretz J. (2010). Node centrality in weighted networks: generalizing degree and shortest paths. *Soc. Netw.* 32 245–251. 10.1016/j.socnet.2010.03.006

[B59] Pfohl B. Coryell W. Zimmerman M. Stangl D. (1986). DSM-III personality disorders: diagnostic overlap and internal consistency of individual DSM-III criteria. *Compr. Psychiatry* 27 21–34. 10.1016/0010-440x(86)90066-0 3948501

[B60] Prigerson H. Wolfson L. Shear M. Hall M. Bierhals A. Zonarich D. (1997). Case histories of traumatic grief. *Omega* 35 9–24.

[B61] Prigerson P. Maciejewski P. Reynolds C. Bierhals A. Newson J. Fasiczka A. (1995). Inventory of complicated grief: a scale to measure maladaptive symptoms of loss. *Psychiatry Res.* 59 65–79. 10.1016/0165-1781(95)02757-2 8771222

[B62] Rhemtulla M. Fried E. Aggen S. Tuerlinckx F. Kendler K. Borsboom D. (2016). Network analysis of substance abuse and dependence symptoms. *Drug Alcohol Depend* 161 230–237. 10.1016/j.drugalcdep.2016.02.005 26898186 PMC4861635

[B63] Roberts N. Roberts P. Jones N. Bisson J. (2015). Psychological interventions for post-traumatic stress disorder and comorbid substance use disorder: a systematic review and meta-analysis. *Clin. Psychol. Rev.* 38 25–38. 10.1016/j.cpr.2015.02.007 25792193

[B64] Robinaugh D. LeBlanc N. Vuletich H. McNally R. (2014). Network analysis of persistent complex bereavement disorder in conjugally bereaved adults. *J. Abnorm. Psychol.* 123 510–522. 10.1037/abn0000002 24933281 PMC4170793

[B65] Robinaugh D. Millner A. McNally R. (2016). Identifying highly influential nodes in the complicated grief network. *J. Abnom. Psychol.* 125 747–757. 10.1037/abn0000181 27505622 PMC5060093

[B66] Robinson T. Marwit S. (2006). An investigation of the relationship of personality, coping, and grief intensity among bereaved mothers. *Death Studies* 30 677–696. 10.1080/07481180600776093 16869060

[B67] Sanchez-Peña J. F. Alvarez-Cotoli P. Rodrıguez-Solano J. (2012). Trastornos psiquiátricos asociados a alcoholismo: seguimiento a 2 años de tratamiento. *Actas Esp Psiquiatrıa* 40 129–135. 22723131

[B68] Schäfer I. Najavits L. (2007). Clinical challenges in the treatment of patients with posttraumatic stress disorder and substance abuse. *Curr. Opin. Psychiatry* 20 614–618. 10.1097/yco.0b013e3282f0ffd9 17921765

[B69] Sher K. Trull T. (2002). Substance use disorder and personality disorder. *Curr. Psychiatry Rep.* 4 25–29. 10.1007/s11920-002-0008-7 11814392

[B70] Sumnall H. Brotherhood A. (2012). *Social Reintegration and Employment: Evidence and Interventions for Drug Users in Treatment.* Luxembourg: EMCDDA.

[B71] Tomarken A. Roth A. Holland J. Ganz O. Schachter S. Kose G. (2001). Examining the role of trauma, personality, and meaning in young prolonged grievers. *Psychooncology* 21 771–777. 10.1002/pon.1983 21557384

[B72] Trull T. Jahng S. Tomko R. Wood P. Sher K. (2010). Revised NESARC personality disorder diagnoses: gender, prevalence, and comorbidity with substance dependence disorders. *J. Pers. Disord.* 24 412–426. 10.1521/pedi.2010.24.4.412 20695803 PMC3771514

[B73] Walcott G. Martin J. Hickling F. (2013). The prevalence of personality disorder in a psychiatric and substance abuse population in jamaica. *West Indian Med. J.* 62 458–462. 10.7727/wimj.2013.078 24756661

[B74] Widiger T. Trull T. (2007). Plate tectonics in the classification of personality disorder: shifting to a dimensional model. *Am. Psychol.* 62 71–83. 10.1037/0003-066x.62.2.71 17324033

[B75] World Health Organization (2018). *International Classification of Diseases for Mortality and Morbidity Statistics (11th Revision).* Geneva: WHO.

[B76] Zhang B. El-Jawahri A. Prigerson H. (2006). Update on bereavement research: evidence-based guidelines for the diagnosis and treatment of complicated bereavement. *J. Pal. Med.* 9 1188–1203. 10.1089/jpm.2006.9.1188 17040157

[B77] Zimmermann P. Firnkes S. Kowalski J. T. Backus J. Siegel S. Willmund G. (2014). Personal values in soldiers after military deployment: associations with mental health and resilience. *Eur. J. Psychotraumatol.* 5:22939. 10.3402/ejpt.v5.22939 24808938 PMC4012073

[B78] Zuckoff A. Shear K. Frank E. Daley D. Seligman K. Silowash R. (2006). Treating complicated grief and substance use disorders: a pilot study. *J. Subst. Abuse Treat.* 30 205–211. 10.1016/j.jsat.2005.12.001 16616164

