# Supplementary material for: Understanding the Relationship Between Complicated Grief Symptoms and Patterns of Personality Disorders in a Substance Users’ Sample: A Network Analysis Approach
Source: Front Psychol. 2020 Oct 30;11:566785. doi: 10.3389/fpsyg.2020.566785 (PMC7673378; doi:10.3389/fpsyg.2020.566785)
Supplement: Supplementary file 1 [file Table_1.DOCX]

**Table 1**. A zero-order correlation table with complicated grief symptoms

| Psychological constructs | Edge weight |  |
| --- | --- | --- |
| Age | -0.03 |  |
| *Pattern of personality disorder* |  |  |
| schizoid | 0.00 |  |
| avoidant | 0.00 |  |
| depressive | 0.15 |  |
| dependent | 0.00 |  |
| histrionic | -0.02 |  |
| narcissistic | -0.02 |  |
| antisocial | -0.06 |  |
| sadistic | 0.00 |  |
| compulsive | 0.00 |  |
| negativistic | 0.00 |  |
| masochistic | 0.00 |  |
| schizotypal | 0.04 |  |
| borderline | 0.05 |  |
| paranoid | 0.12 |  |
